# Adiponectin Assists Thrombopoietic Agents in ITP Treatment by Enhancing Myosin‐9/Rab6A‐Mediated Trafficking of c‐Mpl in MKs

**DOI:** 10.1002/advs.202503008

**Published:** 2025-06-25

**Authors:** Xin Zhao, Huixian Ma, Guosheng Li, Jilong Xiao, Zhiyu Shi, Mingying Li, Xinguang Liu, Tao Sun, Chunyan Ji

**Affiliations:** ^1^ Department of Hematology Qilu Hospital of Shandong University Jinan Shandong Province 250012 China; ^2^ Cryomedicine Laboratory Qilu Hospital of Shandong University Jinan Shandong Province 250012 China; ^3^ Department of Immunology School of Biomedical Sciences Shandong University Jinan Shandong Province 250012 China; ^4^ Innovative Institute of Chinese Medicine and Pharmacy Shandong University of Traditional Chinese Medicine Jinan Shandong Province 250355 China; ^5^ Shandong Key Laboratory of Hematological Diseases and Immune Microenvironment Qilu Hospital of Shandong University Jinan Shandong Province 250000 China

**Keywords:** adiponectin, c‐Mpl, ITP, membrane trafficking, Myosin‐9/Rab6A complex

## Abstract

Immune thrombocytopenia (ITP) is an autoimmune disorder characterized by accelerated platelet destruction and defective platelet production. The thrombopoietin (TPO)‐activated c‐Mpl signaling pathway has been proven to promote megakaryocyte differentiation and platelet production and thus has significant value in the clinical treatment of ITP. However, individual differences and unsustainable responses limit the clinical application of c‐Mpl agonists. The cell membrane distribution of c‐Mpl is crucial for the cell response to c‐Mpl agonists. In the present study, this is observed that the distribution of c‐Mpl on the megakaryocyte membrane is significantly reduced in ITP patients. The reduction is more severe in refractory patients. Then, this is verified that the membrane trafficking of c‐Mpl is mediated by the Myosin‐9/Rab6A complex, and demonstrates that the stability of this complex depends on Rab6A‐GTPase. Furthermore, this is found that adiponectin promotes the membrane localization of c‐Mpl by increasing the expression of Rab6A GEFs. Finally, this is revealed that adiponectin can assist thrombopoietic agents in the treatment of ITP mice. The results clarify the c‐Mpl distribution defects in the cell membrane and the corresponding c‐Mpl membrane transport mechanisms in megakaryocytes of ITP, providing new insights to improve the clinical efficacy of thrombopoietic agents for ITP patients.

## Graphical abstract

This study revealed that adiponectin facilitates the therapeutic efficiency of thrombopoietic agents by stimulating the membrane trafficking of c‐Mpl in MKs, which provides a point for ITP therapy. Moreover, a novel c‐Mpl trafficking mechanism based on the Myosin‐9/Rab6A complex is constructed. These findings provide new insights into the applicability of adiponectin in ITP.

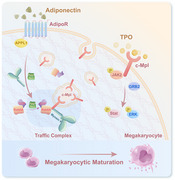

## 1 Introduction

Immune thrombocytopenia (ITP) is an acquired autoimmune disorder characterized by thrombocytopenia with uncertain etiology and is ill‐defined. Accelerated destruction^[^ ^1^, ^2^ ^]^ and suppressed production^[^ ^3^, ^4^ ^]^of platelets are the two main ITP pathogeneses. Due to heterogeneity, the primary goal of therapy for ITP is to maintain the platelet count and prevent bleeding risks through classical treatments (glucocorticosteroids, intravenous immunoglobulins). However, eliciting a long‐term and efficient response in ITP patients remains challenging.^[^ ^5^, ^6^ ^]^ Exploration of the underlying mechanism and discovery of potential therapeutic agents have become popular topics of research for ITP treatment.

Thrombopoietin (TPO) and its receptor c‐Mpl play critical roles in hematopoietic stem cell renewal, megakaryocyte (MK) differentiation, and platelet formation.^[^ ^7^ ^]^ As thrombopoietic agents, rhTPO and TPO‐RAs have been used as widespread therapies for ITP. An effective clinical response has been obtained in some patients. ^[^ ^5^ ^]^ However, 20% of ITP patients fail to achieve a response to c‐Mpl agonists, and most patients rapidly experience relapse and lose their sensitivity to c‐Mpl agonists after drug discontinuation.^[^ ^8^, ^9^ ^]^ The failure mechanism of c‐Mpl agonists urgently needs to be clarified. The membrane distribution of c‐Mpl is crucial for its functional performance. For example, c‐Mpl mutations (R102P, R464G) have been found in congenital amegakaryocytic thrombocytopenia (CAMT) patients.^[^ ^10^, ^11^, ^12^ ^]^ These c‐Mpl mutations were blocked in the endoplasmic reticulum without the ability to translocate to the plasma membrane. In contrast, hyperactivation of TPO or c‐Mpl mutants (S505N, P106L) leads to hereditary thrombocythemia (HT),^[^ ^13^, ^14^ ^]^ which is due to the autonomous dimerization of c‐Mpl and is independent of thrombopoietin homeostasis. All of these reports show that the functional abnormalities of c‐Mpl mutants are related to their abnormal membrane surface distribution, which suggests that the membrane localization of c‐Mpl determines the downstream signaling pathways involved in TPO and MK development. However, there are no reports about the distribution of c‐Mpl on the MK membrane in ITP patients.

The membrane surface distribution of c‐Mpl is affected by many factors. For example, Janus kinase 2 (JAK2), which activates TPO/c‐Mpl signaling via the STAT3, STAT5, and MAPK pathways, enhances cell‐surface localization and stabilizes the mature form of c‐Mpl in MPNs.^[^ ^15^ ^]^ A calreticulin mutant restores the defective cell surface expression of c‐Mpl R464G in CAMT. ^[^ ^12^ ^]^C‐Cbl regulates the trafficking and internalization of c‐Mpl. ^[^ ^7^ ^]^ Transport complexes with molecular motors as their core are crucial for the membrane surface distribution of receptors.^[^ ^16^ ^]^ However, the trafficking complexes and motors that carry c‐Mpl to the cell surface have largely not been explored.

**Scheme 1:**
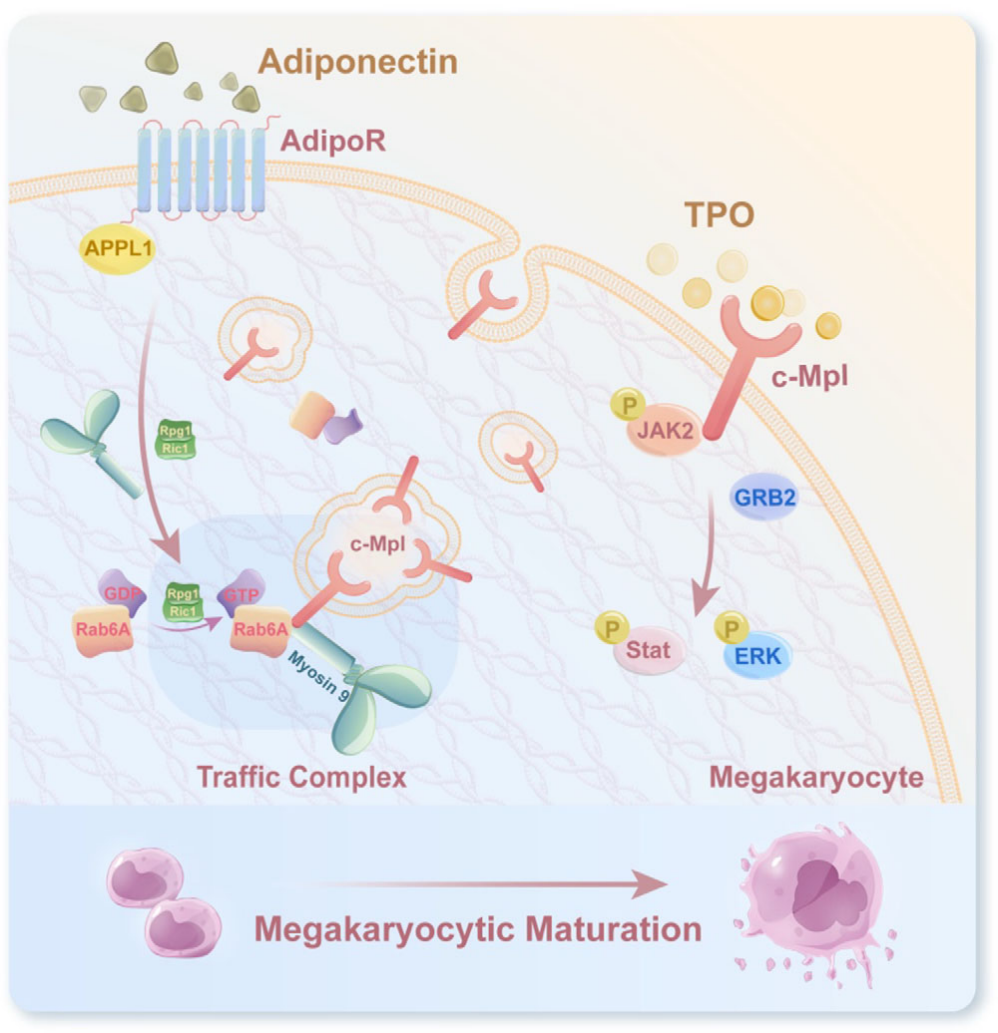
This schematic illustrates the mechanism by which adiponectin enhances the therapeutic efficacy of thrombopoietic agents in ITP. In megakaryocytes (MKs), adiponectin promotes the expression of Rab6A GEFs, which in turn stabilizes the Myosin‐9/Rab6A trafficking complex. This complex facilitates the membrane localization of c‐Mpl, thereby restoring TPO responsiveness and enhancing megakaryocytic maturation.

In this study, we found reduced c‐Mpl cell‐surface expression in MKs from ITP patients and from an ITP murine model. Moreover, Myosin‐9/Rab6A, a newly identified trafficking complex, was proven to transport c‐Mpl to the membrane in a process dependent on GTP‐bound Rab6A. Ultimately, we proved that adiponectin, in combination with thrombopoietic agents, enhanced the combination of GTP‐Rab6A and c‐Mpl, which increased the cell‐surface expression of c‐Mpl and restored the platelet count in ITP mice. In conclusion, this study explored a novel c‐Mpl transport model and demonstrated that the combination of adiponectin with thrombopoietic agents may be an effective strategy for the treatment of ITP (**Scheme** **1**).

## 2 Results

### 2.1 Cell‐surface c‐Mpl Levels are Reduced in ITP MKs

**Figure 1:**
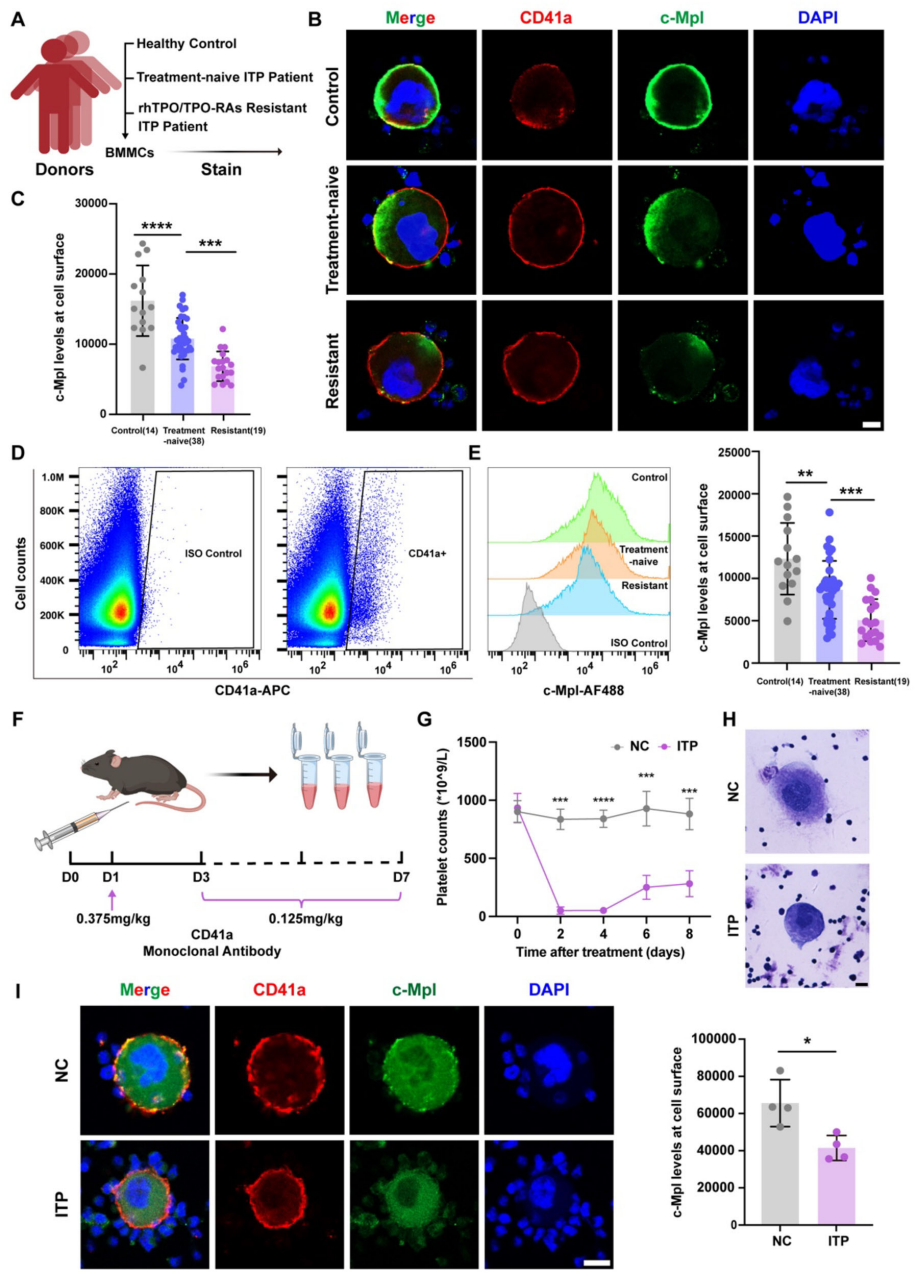
Cell‐surface c‐Mpl levels are reduced in ITP MKs A) Schematic for the acquisition and staining of bone marrow mononuclear cells (BMMCs). B,C) Immunofluorescence (IF) microscopy images (B) and the quantitative summary (C) of c‐Mpl expression on the surface of MKs from treatment‐naïve primary ITP patients (n = 38), rhTPO/TPO‐RAs resistant primary ITP patients (n = 19) and healthy control subjects (n = 14). At least 15 cells were quantified per donor. CD41a, red; c‐Mpl, green. Scale bars, 10 µm. ***P < 0.001; ****P < 0.0001. D) Flow cytometry strategy for the selection of the MK population. E) Representative histograms and statistical analysis for c‐Mpl expression on the surface of MKs from BM samples by flow cytometry. ISO Control represents staining with no primary antibody. The geometric mean fluorescence intensity is shown. **P < 0.01; ***P < 0.001. F) Scheme of the design and procedures of the passive ITP murine model in which was administered CD41a antibody through intraperitoneal injection into C57BL/J mice (6–8 weeks). (G) Platelet counts of peripheral blood from NC and ITP mice were monitored at different time points. (n = 5). ***P < 0.001; ****P < 0.0001. H) Imaging of megakaryocytes stained with Wright–Giemsa stain from the NC and ITP mouse bone marrow samples. I) IF imaging and quantitative summary of the expression of c‐Mpl on the MK surface from the bone marrow of NC and ITP mice. At least 50 cells were quantified per mouse bone marrow (n = 4). CD41a, red; c‐Mpl, green. Scale bars, 10 µm. *P < 0.05. The means ± SDs are shown for the statistical analysis.

As a TPO receptor, c‐Mpl has a critical function in MK maturation and platelet production. Previous work has demonstrated that decreased expression of c‐Mpl on the cell surface of platelets or MKs is a critical feature of myelofibrosis or CAMT; thus, we sought to determine the level of c‐Mpl on the MK membrane in ITP.^[^ ^15^ ^]^ First, we analyzed the bone marrow aspiration from ITP patients and found that the percentage of thromocytogenic megakaryocyte is decreased significantly, whereas the percentage of promegakaryocyte or granular megakaryocyte is increased, which suggested that the development of MK was delayed (Figure S1, Supporting Information). Then, we performed immunofluorescence staining on the surface of MKs from treatment‐naïve or refractory ITP patients and healthy controls. Compared with that in the controls, the cell surface localization of c‐Mpl in the MKs of ITP patients was decreased (**Figure** **1**A–C). Interestingly, we found that the expression of c‐Mpl on the cell surface in MKs from refractory patients was lower than that in MKs from other groups (Figure 1A–C). To further verify this observation, we measured the membrane density of c‐Mpl using FCM, which showed that the MFI of c‐Mpl was significantly lower in the ITP group compared with the control group, which suggested that defective membrane localization of c‐Mpl is a critical cause of ITP (Figure 1D,E). To further address this issue, we constructed a passive ITP mouse model and verified the model by determining platelet counts and performing Wright‐Giemsa staining (Figure 1F–H). Consistent with the results for MKs from patients, lower levels of c‐Mpl were detected on the cell surface in ITP MKs (Figure 1I). These data consistently indicate that the membrane trafficking of c‐Mpl is defective in ITP MKs.

### 2.2 The Myosin‐9/Rab6A Trafficking Complex Transports c‐Mpl to the Cell Membrane

**Figure 2:**
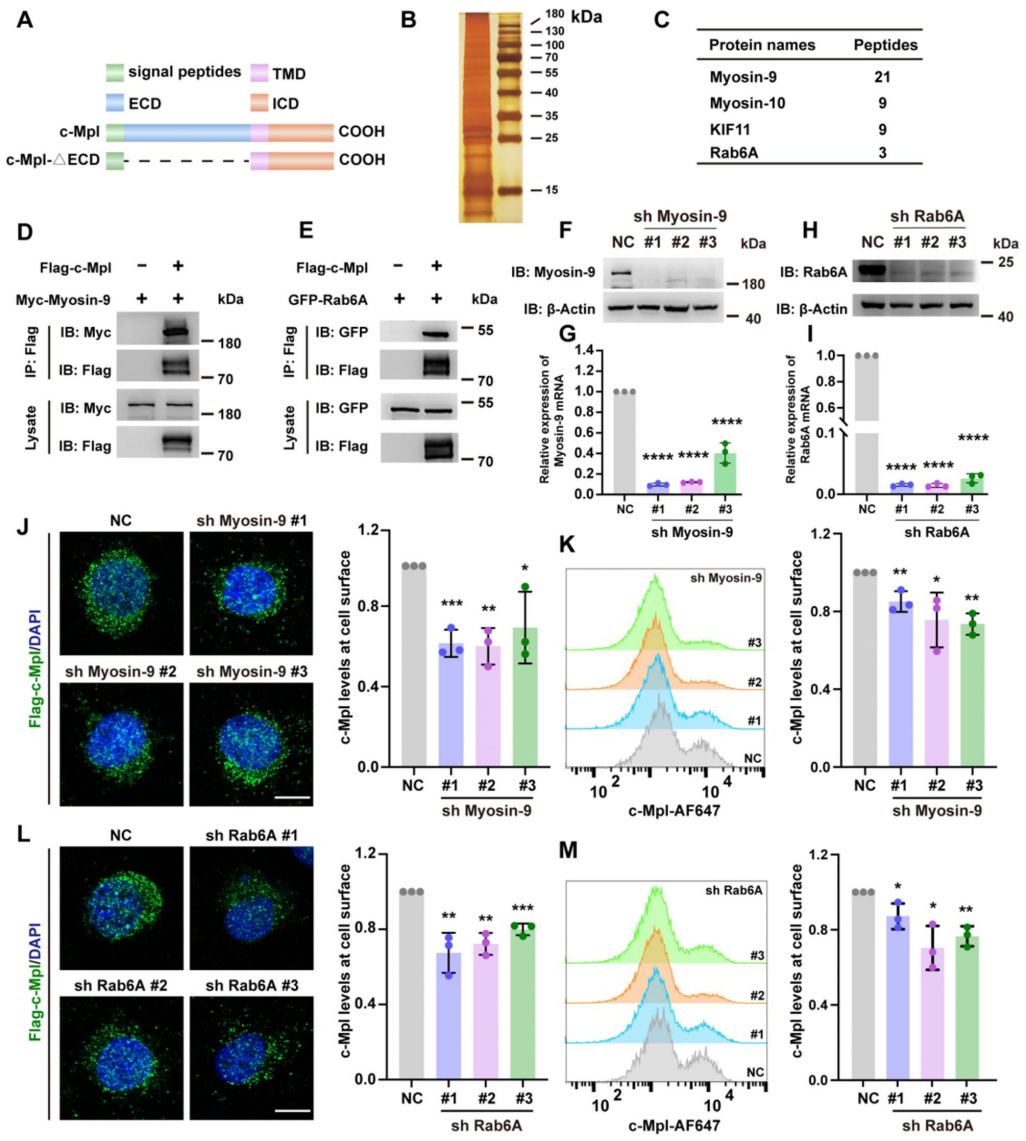
The Myosin‐9/Rab6A trafficking complex transports c‐Mpl to the cell membrane A) An illustration of the structures of c‐Mpl and the c‐Mpl‐C‐terminal domain are presented as cartoons. TMD, transmembrane domain. ECD, extracellular domain. ICD, intracellular domain. B) A silver‐stained SDS gel shows proteins immunoprecipitated to the c‐Mpl‐C‐terminal domain from HEK‐293T cells using an anti‐Flag antibody. C) The top four trafficking‐associated proteins detected by mass spectrometry from the IP gel products. D,E) Immunoblotting shows that Flag‐c‐Mpl does coimmunoprecipitates with Myc‐Myosin‐9 (D) and GFP‐Rab6A (E) in HEK‐293T cells. F,G) The protein expression (F) and relative mRNA expression (G) of Myosin‐9 in shMyosin‐9 UT‐7 cells (n = 3). ****P < 0.0001. H,I) The protein expression (H) and relative mRNA expression (I) of Rab6A in shRab6A UT‐7 cells (n = 3). ****P < 0.0001. J,L) IF images and normalized quantitative analysis of the expression of c‐Mpl on the cell surface of shMyosin‐9 (J) and shRab6A (L) UT‐7 cells. At least 30 cells were quantified per individual experiment (n = 3). c‐Mpl, green. Scale bars, 10 µm. *P < 0.05; **P < 0.01; ***P < 0.001. K,M) Representative histograms and statistical analysis of c‐Mpl expression on the surface of shMyosin‐9 K) and shRab6A (M) UT‐7 cells determined via flow cytometry. The normalized geometric mean fluorescence intensity is shown (n = 3). *P < 0.05; **P < 0.01. All of the UT‐7 cells were transduced with Flag‐c‐Mpl. The means ± SDs are shown.

The mechanism of c‐Mpl trafficking in MKs is poorly understood. To identify potential regulators of c‐Mpl trafficking, we constructed a plasmid containing the c‐Mpl C‐terminal domain and sorted the associated proteins by Co‐IP (**Figure** **2**A,B). The proteins were detected by mass spectrometry, and the top four trafficking‐associated proteins were identified, namely: Myosin‐9, Myosin‐10, Kif11, and Rab6A (Figure 2C). Based on our interest in submembrane proteins, compared to Kifs that walk on the tubulin cytoskeleton, myosins, which are associated with the actin pathway, attracted our attention. Furthermore, a previous study reported that the expression of Myosin‐9 is increased during MK differentiation, while that of Myosin‐10 is decreased.^[^ ^17^ ^]^ Therefore, Myosin‐9 is an important topic of study. In addition, as a general regulator of cargo trafficking, Rab6A controls some physiological and pathological activities^[^ ^18^ ^]^ and has the potential to regulate c‐Mpl transport. Therefore, the interaction between c‐Mpl and Myosin‐9 or Rab6A was verified by Co‐IP (Figure 2D,E). Then, Myosin‐9 or Rab6A was knocked down in UT‐7 cells, and the protein and mRNA levels of Myosin‐9 and Rab6A were confirmed to be markedly reduced compared with those in the control group (Figure 2F,I). Immunofluorescence and FCM results showed that Myosin‐9 deficiency resulted in lower c‐Mpl levels on the cell surface of c‐Mpl‐transduced UT‐7 cells, (Figure S2,Supporting Information; Figure 2J,K), indicating that Myosin‐9 participates in c‐Mpl trafficking to the cell surface. The trafficking of c‐Mpl was also defective in Rab6A‐deficient cells (Figure 2L,M). These results indicate that the trafficking of c‐Mpl requires the pattern of Myosin‐9 and Rab6A.

### 2.3 The Formation of Myosin‐9/Rab6A Trafficking Complex is Dependent on Rab6A GTPase and Enhances MK Maturation via the TPO/c‐Mpl Signaling Pathway

**Figure 3:**
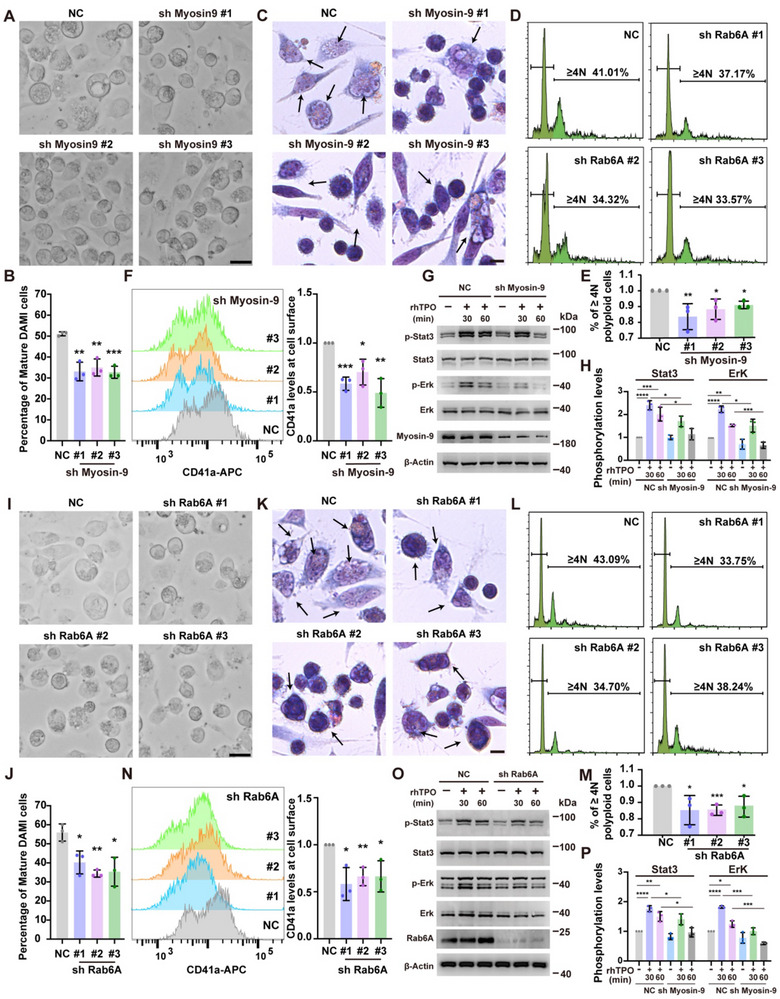
Myosin‐9/Rab6A trafficking complex enhances MK maturation via the TPO/c‐Mpl signaling pathway A,B) Photomicrograph showing morphological differences (cell size) in NC and shMyosin‐9 DAMI cells after 72 h of PMA treatment. Scale bars, 20 µm. Quantitative analysis of the percentage of mature DAMI cells (Feret's diameter ≥20 µm) is shown. At least 30 cells were quantified per individual experiment (n = 3). **P < 0.01; ***P < 0.001. C) Imaging of PMA‐treated NC and shMyosin‐9 DAMI cells stained with Wright–Giemsa. The black arrows show morphological mature DAMI cells with multiple nuclei. Scale bars, 20 µm. D,E) Detection of DNA content (≥4 N) in PMA‐treated NC and shMyosin‐9 DAMI cells (n = 3). ****P < 0.0001. F) Representative histograms and statistical analysis of CD41a expression in PMA‐treated NC and shMyosin‐9 DAMI cells determined by flow cytometry. The normalized geometric mean fluorescence intensity is shown (n = 3). *P < 0.05; **P < 0.01; ***P < 0.001. G,H) Western blot assays were conducted to analyze the expression of the Stat3/Erk pathway from TPO/c‐Mpl signaling in PMA‐treated NC and shMyosin‐9 DAMI cells. Quantitative analysis of p‐Stat3/p‐Erk is shown. *P < 0.05; **P < 0.01; ***P < 0.001; ****P < 0.0001. I‐P) Same as in Figure 3A–H but with shRab6A instead of shMyosin‐9 DAMI cells. The data are shown as the mean ± SD of three independent experiments.

To explore the function of Myosin‐9/Rab6A in the MK maturation process, Myosin‐9/Rab6A‐deficient DAMI cells were constructed, and the interference efficiency was verified by RT‒PCR and Western blotting (Figure S3A–F, Supporting Information). Previous studies have suggested that PMA stimulates the differentiation of DAMI cells; therefore, the use of PMA is a tool for exploring the function and mechanism associated with MK differentiation.^[^ ^19^, ^20^ ^]^ To further substantiate the role of Myosin‐9/Rab6A in MK differentiation, we treated DAMI cells with PMA (100 nM) and examined MK‐related morphological features, cell surface antigens, and polyploidy. Regarding morphological features, we found that the percentage of mature DAMI cells (Feret's diameter ≥20 µm) was decreased in Myosin‐9/Rab6A‐deficient cells, which suggested that Myosin‐9/Rab6A deficiency impaired the differentiation of DAMI cells (**Figure** **3**A,B,I–J). The incidence of polyploidy in cells with Myosin‐9 or Rab6A deficiency was also decreased significantly (Figure 3C–E,K–M). CD41a plays important roles in platelet acceleration and aggregation, and the expression of CD41a is now used as a maturation marker of megakaryocytes.^[^ ^21^ ^]^ We found that the number of CD41a‐positive cells was significantly decreased in Myosin‐9/Rab6A‐deficient cells compared to control cells (Figure 3F,N). It is known that MK development is dependent on the TPO/c‐Mpl signal transducer and Stat3/Erk signaling pathway. To elucidate the regulatory function of Myosin‐9/Rab6A in TPO/c‐Mpl signaling, the activity of the Stat3/Erk pathway was measured. The results showed that the signaling activity was lower in the gene knockdown group than in the control group (Figure 3G,H,O,P). These results confirmed that the Myosin‐9/Rab6A trafficking complex is needed for MK maturation.

**Figure 4:**
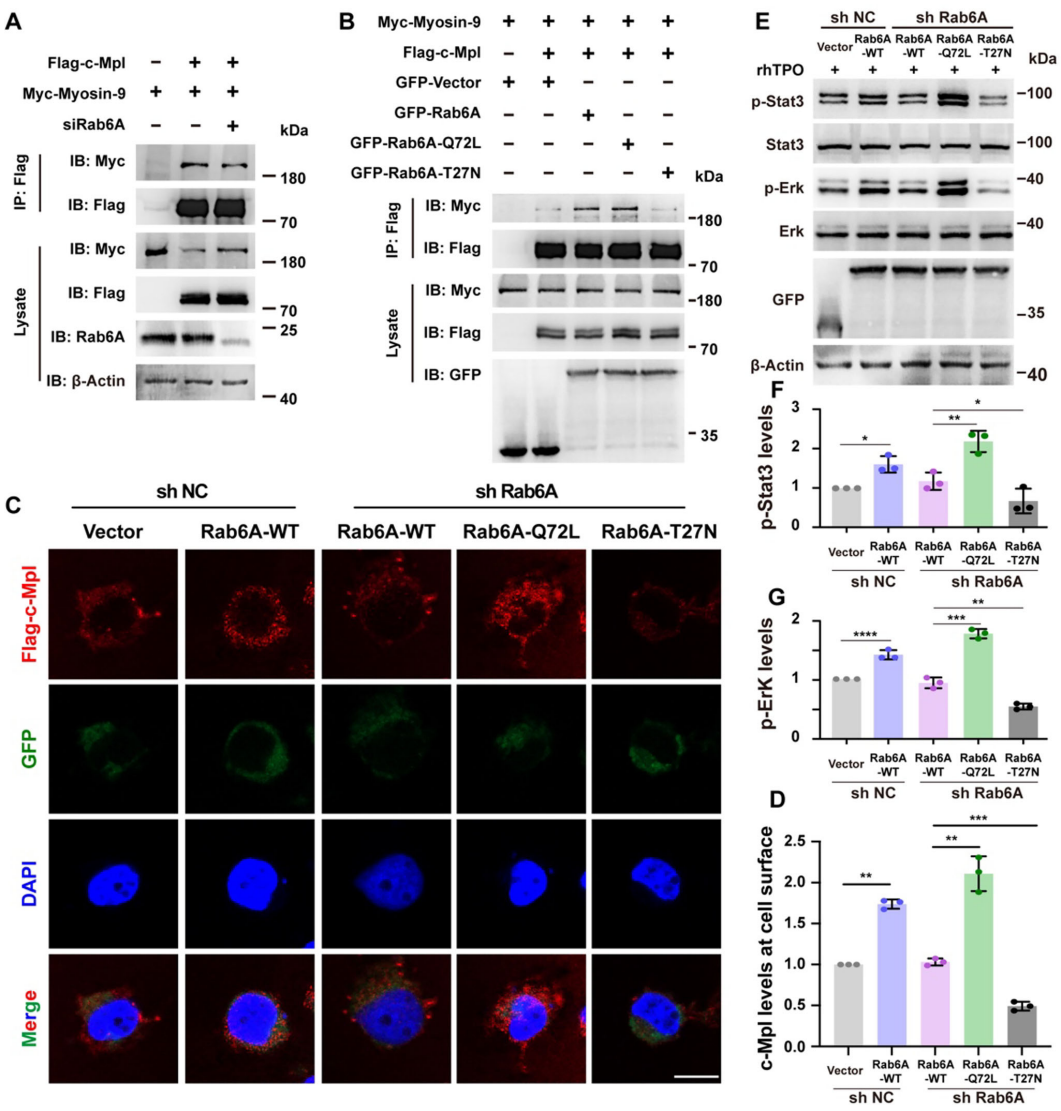
The formation of the Myosin‐9/Rab6A trafficking complex is dependent on Rab6A GTPase A) Co‐IP immunoblotting shows that the coimmunoprecipitation of Myc‐Myosin‐9 and Flag‐c‐Mpl was destroyed by Rab6A interference in HEK‐293T cells. B) Co‐IP of Myc‐Myosin‐9 and Flag‐c‐Mpl immunoblotting shows that Rab6A‐Q72L promotes the coprecipitation of c‐Mpl with Myosin‐9, while Rab‐T27N does opposite. C,D) Immunofluorescence staining (C) and normalized quantitative analysis (D) of c‐Mpl expression on the surface of the indicated UT‐7 cells. At least 30 cells were quantified per individual experiment (n = 3). c‐Mpl, red; GFP, green. Scale bars, 10 µm. *P < 0.05; **P < 0.01; ***P < 0.001. E‐G) Western blot assays were conducted to analyze the expression of the Stat3/Erk pathway in the indicated HEK‐293T cells with rhTPO added. Quantitative analysis of p‐Stat3/p‐Erk is shown. *P < 0.05; **P < 0.01; ***P < 0.001; ****P < 0.0001. The means ± SDs are shown for the statistical analysis.

As expected, according to the immunoprecipitation assay, the interaction between Myosin‐9 and c‐Mpl was abolished by Rab6A interference (**Figure** **4**A). A large group of Rabs play important roles in regulating the membrane trafficking system; specifically, they exchange the inactive GDP‐bound form with an active GTP‐bound form.^[^ ^22^, ^23^ ^]^ Thus, Rab6A‐Q72L, a constitutively active GTPase Rab6A mutant, and Rab‐T27N, a GDP‐bound Rab6A mutant, were constructed. The results showed that Rab6A‐Q72L promoted c‐Mpl Co‐IP with Myosin‐9 and that Rab‐T27N did not activate c‐Mpl/Myosin‐9 (Figure 4B), suggesting that the transport of c‐Mpl was mediated by Rab6A GTPase activity. Therefore, the function of Rab6A GTPase activity in c‐Mpl transport and MK maturation was studied. Notably, the level of c‐Mpl on the cell surface was increased when Rab6A‐Q72L was overexpressed, but the level was decreased in the Rab‐T27N group (Figure 4C,D). Furthermore, Rab6A‐Q72L eliminated the disruption of signaling activity caused by Rab6A deficiency, but the activating effect of Rab‐T27N was limited (Figure 4E–G). These observations were consistent with the Co‐IP results and indicated that GTP‐bound Rab6A promoted c‐Mpl transport on the cell surface by regulating the binding of Myosin‐9 and c‐Mpl.

### 2.4 Adiponectin Promotes the Distribution of c‐Mpl on Cell Membranes via Enhancing the Expression of Rab6A GEFs

**Figure 5:**
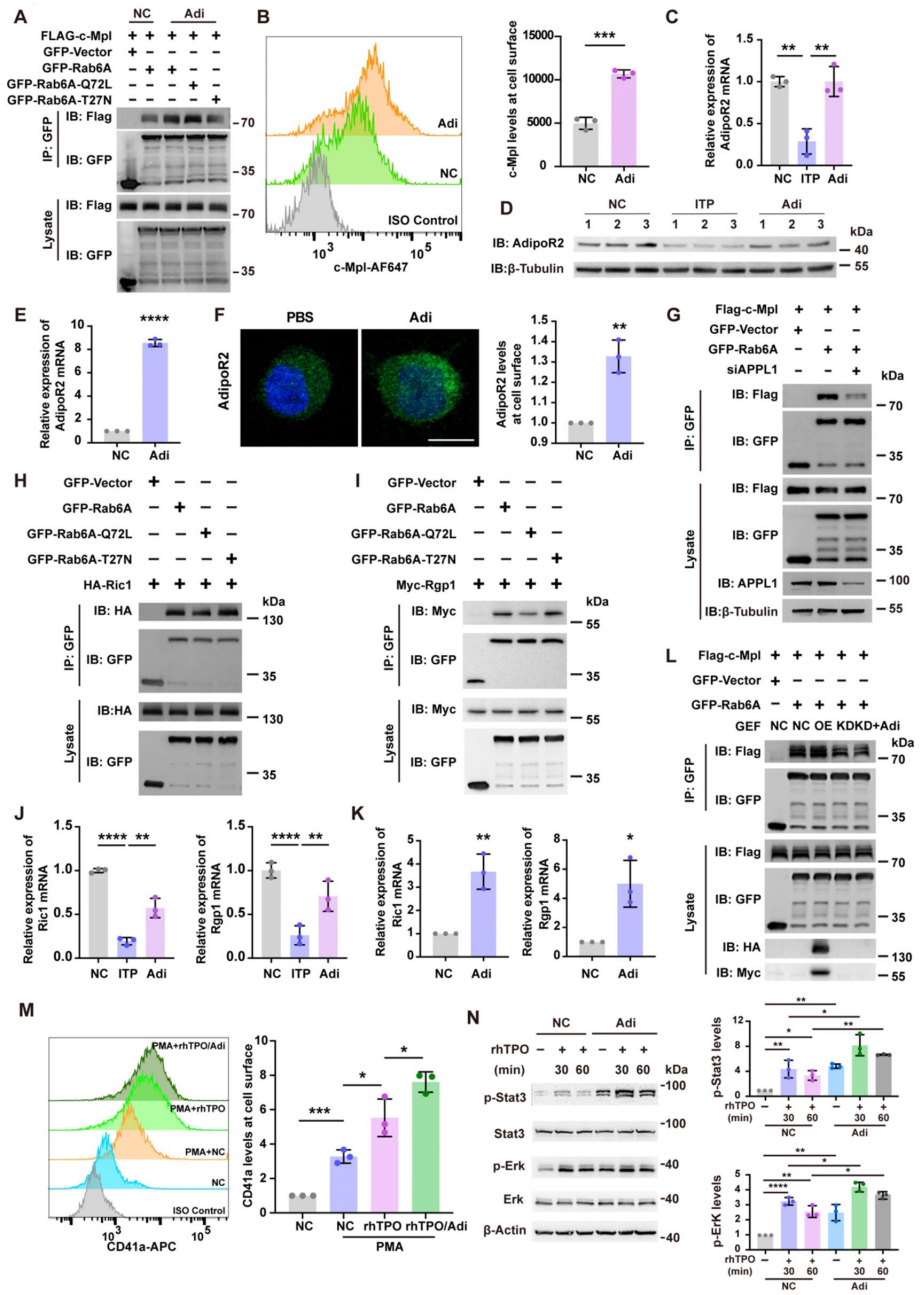
Adiponectin promotes the distribution of c‐Mpl on cell membranes via enhancing the expression of Rab6A GEFs A) Co‐IP immunoblotting of GFP‐Rab6A and Flag‐c‐Mpl shows that the coimmunoprecipitation of c‐Mpl and Rab6A is promoted by adiponectin (Adi). B) Representative histograms and statistical analysis of c‐Mpl expression on surface of NC and adiponectin treated UT‐7 cells determined via flow cytometry. The geometric mean fluorescence intensity is shown(n = 3). ***P < 0.001. C,D) The relative mRNA expression (C) and protein expression (D) of adiponectin receptor 2 (AdipoR2) from the mice bone marrow. (n = 3). **P < 0.01. E) The relative mRNA expression of AdipoR2 in the DAMI cells after the adiponectin treatment. ****P < 0.0001. F) IF images and the normalized quantitative analysis of the expression of AdipoR2 on cell surface from the adiponectin treated DAMI cells. At least 30 cells were quantified per individual experiment (n = 3). AdipoR2, green. Scale bars, 10 µm. **P < 0.01. G) Co‐IP immunoblotting of Flag‐c‐Mpl with GFP‐Rab6A shows that the coimmunoprecipitation of c‐Mpl and Rab6A was down‐regulated by siAPPL1. H,I) Co‐IP immunoblotting of HA‐Ric1(H) and Myc‐Rgp1(I) with GFP‐Rab6A and GFP‐Rab6A mutants shows that the Ric1 and Rgp1 bind to Rab6A‐T27N. J) The mRNA relative expression of Ric1 and Rgp1 in the mice bone marrow. (n = 3). **P < 0.01; ****P < 0.0001. K) The mRNA relative expression of Ric1 and Rgp1 in the DAMI cells after the adiponectin treatment. (n = 3). *P < 0.05; **P < 0.01. L) Co‐IP immunoblotting of Flag‐c‐Mpl with GFP‐Rab6A shows that overexpression (OE) of GEF promoted the binding of c‐Mpl to Rab6A, whereas knockdown (KD) of GEF decreases the binding. Overexpression or knockdown of GEF represents simultaneous overexpression or knockdown of Ric1 and Rgp1. M) Representative histograms and statistical analysis for CD41a expression of indicated DAMI cells by flow cytometry. Normalized geometric mean fluorescence intensity is shown(n = 3). *P < 0.05; ***P < 0.001. N) Western blot assays were conducted to analyze the expression of Stat3/Erk pathway from TPO/c‐Mpl signaling in PBS or adiponectin treated DAMI cells. Quantitative analysis of p‐Stat3/p‐Erk was shown. *P < 0.05; **P < 0.01; ***P < 0.001; ****P < 0.0001. Data are shown as mean ± SD of three independent experiments.

Adiponectin, which is secreted from adipocytes, stimulates receptor membrane translocation by small GTPases, such as glucose transporter 4 (Glut4),^[^ ^24^ ^]^ which suggests that adiponectin stimulates the interaction between Rab6A and c‐Mpl. As expected, the interaction between Rab6A and c‐Mpl was greater in the adiponectin‐treated group than in the control group (**Figure** **5**A). Furthermore, the Co‐IP assay showed that the interaction of Rab6A‐Q72L with c‐Mpl was greater than that of the wild type. In contrast, Rab‐T27N abolished the stimulatory effect of adiponectin (Figure 5A). Next, we measured the change in the level of c‐Mpl on the cell surface after adding adiponectin using immunofluorescence staining. As shown in Figures 5B and S6 (Supporting Information), adiponectin promoted the surface localization of c‐Mpl in a control manner. These results provide strong evidence that the membrane translocation of c‐Mpl is promoted by adiponectin and depends on the active GTPase Rab6A. The controlled and saturable regulation also supports the safety of adiponectin as a modulator in this context.

A previous study has reported that adiponectin stimulates megakaryopoiesis and the expression of the adiponectin receptor was gressively induced.^[^ ^25^ ^]^ However, the elaborate mechanism by which adiponectin regulates Rab6A/c‐Mpl complex transport to the membrane remains unclear. So first, we demonstrated that adiponectin receptor 2 is highly expressed in MKs, including in the DAMI and UT‐7 cell lines (Figure S4A, Supporting Information), and found that the expression of adiponectin receptor 2 was decreased in the MKs of ITP mice than in those of control mice. Importantly, adiponectin rescued these changes (Figure 5C,D). Meanwhile, the expression of adiponectin receptor 2 was markedly elevated after the DAMI cells were treated with adiponectin (Figure 5E,F). To further explore the function of adiponectin in regulating the formation of the Rab6A/c‐Mpl complex, we examined the interaction of the Rab6A/c‐Mpl trafficking complex after knocking down APPL1, a key element in the intracellular signaling of adiponectin (Figure S4B,C,Supporting Information; Figure 5G). The Co‐IP results revealed that the Rab6A/c‐Mpl complex was destroyed by knocking down APPL1. These results illustrate that the formation of the Rab6A/c‐Mpl trafficking complex is regulated by the adiponectin‐receptor 2‐APPL1 signaling axis, which further suggests that adiponectin plays critical roles in TPO/c‐Mpl signaling transduction in MKs.

Next, we explored how adiponectin modulates the formation of trafficking complexes. The activated level of Rabs needs specific regulators: the guanine nucleotide exchange factors (GEFs) to promote Rab's activation, and the GTPase‐activating proteins (GAPs) to terminate Rab's activity.^[^ ^26^ ^]^ Previous studies have identified that Ric1‐Rgp1 complex are the GEFs of Rab6A in Yeast and HEK293T cells.^[^ ^27^, ^28^ ^]^ As expected, we found that Ric1 or Rgp1 strongly interacts with Rab6A‐T27N, GDP‐bound Rab6A; while has a weaker interaction with Rab6A‐Q72L than with Rab6A‐wide type (Figure 5H,I). Interestingly, we found that the expression of Ric1 or Rgp1 decreased significantly in ITP mice but that this change was reversed by adiponectin (Figure 5J). Furthermore, the level of Ric1 or Rgp1 in DAMI cells also increased after the cells were treated with adiponectin (Figure 5K). These results suggested that adiponectin plays an important role in promoting the expression of Rab6A GEFs. To investigate whether adiponectin regulates the Rab6A/c‐Mpl trafficking complex via the adiponectin‐GEF signaling axis, we performed Co‐IP assays and found that GEFs co‐overexpression enhanced the interaction of Rab6A/c‐Mpl, whereas GEFs knockdown disrupted this interaction. Furthermore, the activating effect of adiponectin were disappeared when GEFs were knocked down (Figure S4D,Supporting Information; Figure 5L). These results illustrated that adiponectin promoted Rab6A/c‐Mpl complex formation by enhancing the expression of Rab6A GEFs.

To investigate the effects of adiponectin on the development of MK, we first measured the change in the level of c‐Mpl on the cell surface after adding adiponectin and rhTPO using Flow cytometry (Figure 5M). As shown in Figure 5M, adiponectin increased the efficiency of rhTPO in promoting MK development. Furthermore, TPO/c‐Mpl signaling was strongly stimulated by treatment with adiponectin (Figure 5N). These results provide strong evidence that the membrane translocation of c‐Mpl is promoted by adiponectin and depends on the active GTPase Rab6A by promoting the expression of Rab6A GEFs.

### 2.5 Combining Adiponectin with rhTPO or TPO‐RA Increases the Therapeutic Efficacy of Thrombopoietic Agents in ITP Mice

**Figure 6:**
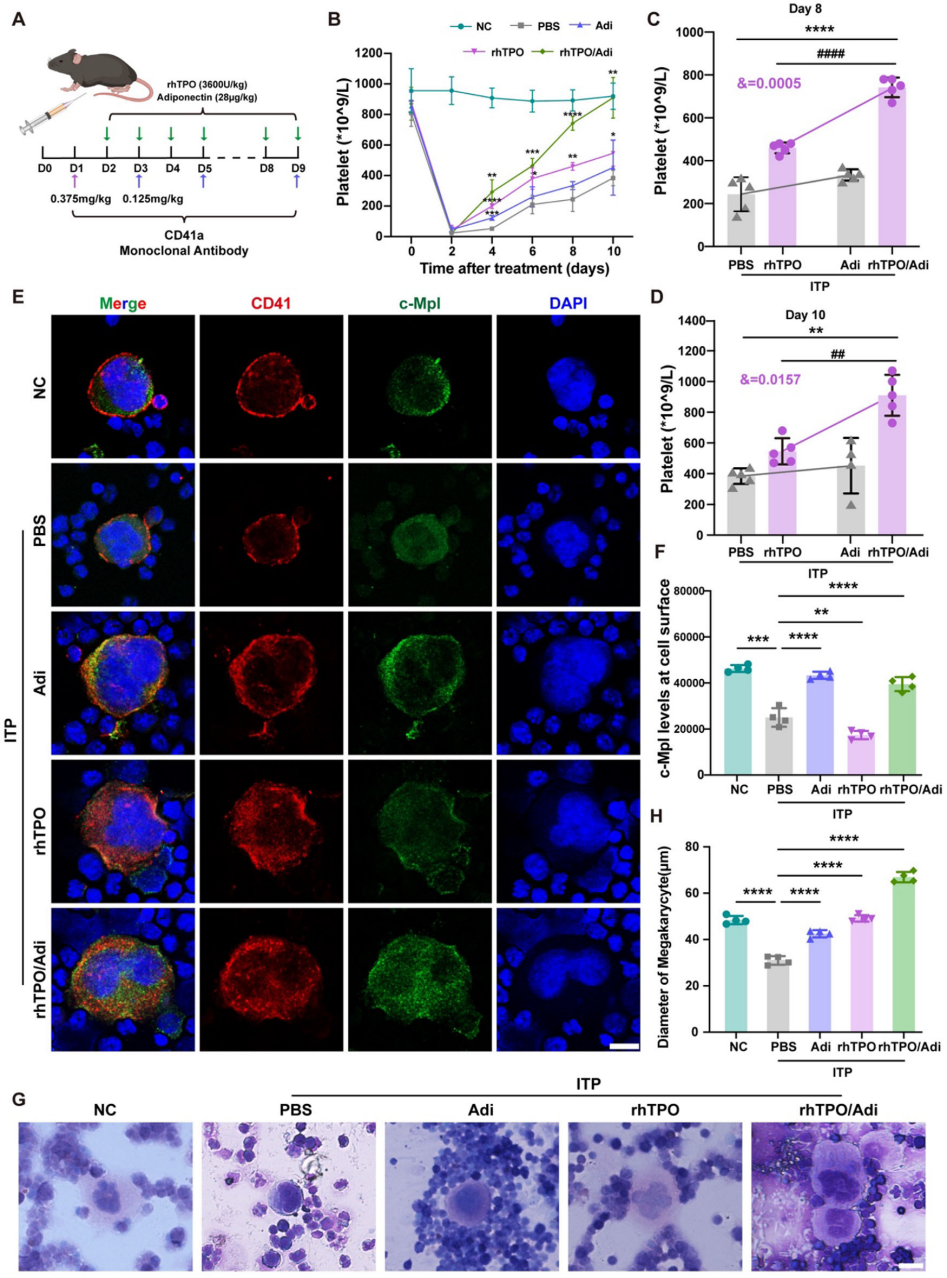
Adiponectin combined with rhTPO increases therapeutic efficacy in ITP mice. A) Schematic outline of the experimental strategy of ITP mice is shown. B) Peripheral blood platelet counts of mice subjected to different treatments were monitored at different time points (n = 5). One‐way ANOVA. **P < 0.01; ***P < 0.001; ****P < 0.0001. C,D) Platelet counts at day 8 (C) and day 10 (D). **P < 0.01; ****P < 0.0001. ##P < 0.01, ####P < 0.0001, versus the PBS group treated with rhTPO and adiponectin; &, interaction effect; two‐way ANOVA. E) IF confocal microscopy images show the expression of c‐Mpl on the MK surface in the bone marrow of mice subjected to different treatments. CD41a, red; c‐Mpl, green. Scale bars, 10 µm. F) Statistical analysis of c‐Mpl expression on the surface of mouse MKs (n = 4). **P < 0.01; ***P < 0.001; ****P < 0.0001. G) Imaging of megakaryocytes stained with Wright–Giemsa from mouse bone marrow. H) Quantitative analysis of the mice MK diameter. At least 30 cells were quantified per mouse bone marrow (n = 4). ****P < 0.0001. The means ± SDs are shown for the statistical analysis.

To confirm whether adiponectin restores platelet production, we applied adiponectin and/or a thrombopoietic agent to ITP mice. As shown in **Figure** **6**A, the platelet counts were increased after the injection of adiponectin or rhTPO. Interestingly, the combination of adiponectin and rhTPO had increased efficacy in treating ITP (Figure 6B–D). Consistent with rhTPO, one kind of TPO‐RA, Romiplostim, also promoted thrombocytopoiesis when combined with adiponectin (Figure S5A, Supporting Information). Moreover, the membrane surface distribution of the c‐Mpl cells was significantly increased in the combination treatment group, which is supported by the in vitro results (Figure 6E,F; Figure S5B, Supporting Information). Subsequently, Wright‐Giemsa staining revealed that the proportion of mature MKs in the rhTPO/adiponectin group was greater than that in the rhTPO‐ and adiponectin‐only groups (Figure 6G,H). These results indicated that combined treatment with adiponectin and rhTPO/Romiplostim could be a novel therapy for ITP.

## 3 Discussion

ITP is a complex and heterogeneous autoimmune disease that is characterized by accelerated platelet clearance and impaired platelet production.^[^ ^29^ ^]^ At present, as widespread therapies, rhTPO and TPO‐RAs are aimed at promoting MK development and platelet production but also have several side effects and limited efficacy.^[^ ^30^ ^]^ Most research demonstrated that baseline TPO level was inversely correlated with response to TPO‐RAs therapy.^[^ ^8^, ^31^ ^]^ In this study, we point to the TPO receptors on the megakaryocyte membrane surface are key determinants of the extent to which patients respond to analogues. We found that the expression of c‐Mpl on the cell surface was lower in ITP patients and in the ITP murine model. Then, we identified a novel trafficking complex that carries c‐Mpl to the cell surface. Finally, we confirmed that the combination of adiponectin with thrombopoietic agents promotes the transport of c‐Mpl to the cell surface by enhancing the expression of Rab6A GEFs, which might be a clinical treatment for alleviating thrombocytopenia.

First, we found that regulating the expression of c‐Mpl on the cell surface in MKs was a potential therapeutic strategy for ITP. As a TPO membrane receptor, c‐Mpl has been proven to be involved in hematopoietic stem cell development, megakaryocyte differentiation, and platelet production.^[^ ^10^ ^]^ The abnormal expression of c‐Mpl on the cell surface is an established feature of several diseases (CAMT, HT, myeloproliferative neoplasms, and others),^[^ ^12^, ^13^, ^15^ ^]^ but there is no reported evidence on the status of c‐Mpl on the MK cell surface in ITP. Notably, the serum TPO concentration and total expression of c‐Mpl are invariably greater in ITP patients than in healthy controls, which contradicts the presence of TPO/c‐Mpl signal transduction defects in ITP.^[^ ^32^ ^]^ In our study, we found decreased cell surface expression of c‐Mpl in the MKs of ITP patients and in a murine model, which illustrated that evaluating the membrane location of c‐Mpl in MKs is necessary for treating ITP patients, especially refractory patients. Moreover, previous studies have suggested that both megakaryocytes and the platelets have been shown to play immunoregulatory roles.^[^ ^33^ ^]^ Megakaryocytes express immune‐sensing receptors, including toll‐like receptors (TLRs) and Fc‐γ receptors, and can modulate the function of T cells and macrophages through surface molecules. Therefore, it is plausible that insufficient c‐Mpl membrane location contributes to immune dysregulation in ITP by obstructing the differentiation and maturation of megakaryocytes. Our results also complement the speculation on the mechanism of whole blood transcriptomic MPL axis integrity^[^ ^34^ ^]^ in eltrombopag non‐responders.

Second, we identified a novel trafficking complex Myosin‐9/Rab6A, that mediates the activity of the TPO/c‐Mpl signaling pathway. Membrane trafficking is essential for the localization of signaling receptors and is associated with many diseases.^[^ ^35^ ^]^ Prior studies have confirmed that different kinds of c‐Mpl mutations result in deficient transport localization.^[^ ^36^, ^37^ ^]^ In addition, the intracellular trafficking of c‐Mpl is mediated by JAK2, C‐Cbl, and calreticulin.^[^ ^7^, ^38^, ^39^ ^]^ However, the cargo trafficking is dependent on motor proteins and their adaptor proteins, and the vehicles that transport c‐Mpl to the membrane have not been reported. In our study, we screened and verified that the Rab6A/Myosin‐9 complex regulates the membrane trafficking of c‐Mpl. Accurately, a previous study has indicated that Myosin‐9 or Rab6A play important roles in MK development. For instance, the expression level of Myosin‐9 is increased during megakaryocyte maturation, and Myosin‐9 mutation‐related disease leads to macrothrombocytopenia.^[^ ^40^, ^41^ ^]^ Rab6A has been shown to regulate cargo trafficking between the Golgi and the membrane.^[^ ^42^ ^]^ Myosin‐9 can bind to GTP‐Rab6A after EtOH treatment.^[^ ^43^ ^]^ Our study revealed that Rab6A/Myosin‐9 regulates MK maturation by promoting the membrane trafficking of c‐Mpl, which deepens our understanding of these previous works and provides novel ideas for the treatment of c‐Mpl‐associated diseases.

Finally, we found that adiponectin enhanced the treatment efficacy of thrombopoietic agents in ITP by promoting the combination of Rab6A and c‐Mpl. Adiponectin, which is secreted from adipocytes, plays critical roles in several metabolic processes, such as obesity, type 2 diabetes, and metabolic syndrome.^[^ ^44^ ^]^ It has been reported that the expression of many membrane receptors, including the adiponectin receptor, is upregulated during MK development.^[^ ^45^ ^]^ Meanwhile, a recent study showed the regulation of platelet function by adiponectin receptor agonists.^[^ ^46^ ^]^ In addition, adiponectin stimulates megakaryopoiesis.^[^ ^47^ ^]^ Some studies also suggest that adiponectin may also alleviate platelet destruction by inhibiting the activation of M1 macrophages and promoting the polarization of M2 macrophages.^[^ ^48^, ^49^ ^]^ As discussed above, emerging evidence reported that c‐Mpl localized on the megakaryocyte membrane regulated immune regulatory processes by affecting the differentiation and maturation of megakaryocytes and adiponectin also exert immunomodulatory effects. These findings suggest that the application of adiponectin may also play a certain role in correcting the immune system of ITP patients. These studies all confirmed that treatment with adiponectin might be a novel approach for treating ITP. Our study reveals the specific mechanism of adiponectin‐assisted treatment of ITP, which will provide a good foundation for the clinical application of adiponectin.

At present, the clinical application of adiponectin is still in the preclinical stage. As an adjunct therapy, adiponectin may bring systemic effects due to its pleiotropic metabolic and immunological actions.^[^ ^50^, ^51^, ^52^, ^53^ ^]^ Its pharmacokinetics, tissue targeting, and dose‐dependent immune effects require further investigation. Meanwhile, TPO‐RAs have shown some adverse events in the treatment of ITP, including thrombotic complications, bone marrow reticulin deposition, and other common side effects such as headache, arthralgia, myalgia, and dizziness.^[^ ^4^, ^54^ ^]^ Although adiponectin enhances the sensitivity of megakaryocytes to TPO‐RA, we believe that future clinical translation for combining adiponectin with thrombopoietic agents must pay attention to their potential side effects, such as safety profiles, optimal dosing, and potential immunologic or metabolic interactions which require validation in larger studies with longer follow‐up periods.

In conclusion, our studies revealed that adiponectin facilitates the therapeutic efficiency of thrombopoietic agents by stimulating the membrane trafficking of c‐Mpl in MKs, which provides a point for ITP therapy. Moreover, a novel c‐Mpl trafficking mechanism based on the Myosin‐9/Rab6A complex was constructed. These findings provide new insights into the applicability of adiponectin in ITP.

## 4 Experimental Section

### Patients and Controls

A total of 38 treatment‐naïve primary ITP patients (21 females and 17 males; age range 19–75 years, median 47 years; platelet count range 0–28×10^9^ L^−1^, median 3×10^9^ L^−1^; Table S1, Supporting Information) and 19 rhTPO/TPO‐RA‐resistant ITP patients (11 females and 8 males; age range 26–80 years, median 56 years; platelet count range 0–23×10^9^ L^−1^, median 3×10^9^ L^−1^; Tables S1 and S2, Supporting Information) were enrolled in the Department of Hematology, Qilu Hospital, Shandong University, from 2021‐01 to 2023‐10. Adults with the diagnosis of primary ITP, according to the previously international consensus report,^[^ ^55^ ^]^ were required to be aged ≥ 17 years and to have a baseline platelet count < 30×10^9^ L^−1^. For the enrollment of rhTPO/TPO‐RA‐resistant ITP patients, the criteria were that they had been given corticosteroids and that at least one of the treatments was rhTPO injection or TPO‐RAs. Healthy bone marrow was obtained from 14 donors after bone marrow transplantation (5 females and 9 males; age range 23–56 years, median 41.5 years; platelet count range 187–291×10^9^ L^−1^, median 231×10^9^ L^−1^; Table S1, Supporting Information). This study was approved by the Ethics Committee of Qilu Hospital, Shandong University. All patients and healthy donors provided informed consent in accordance with the Declaration of Helsinki.

### Passive ITP Murine Model Establishment and Treatment

Based on previously published methods, a passive ITP murine model was established using male C57BL/6 mice (6‐8 weeks of age, ≈20 g), which were purchased from Vital River Laboratories. Briefly, the platelet counts of all the mice were measured before an anti‐CD41a antibody (clone: MWReg30; BioLegend Cat. No. 133 940) was administered to the ITP groups at 0.375 mg kg^−1^ by intraperitoneal injection on day 1. ITP mice were injected with 0.125 mg kg^−1^ CD41a antibody each other day beginning on day 3. To explore the effect of rhTPO and adiponectin, beginning on day 2, when the platelet counts reached their nadir, the mice were administered 3600 U kg^−1^ rhTPO by hypodermic injection and 28 µg kg^−1^ adiponectin by intraperitoneal injection for 8 consecutive days. To explore the effect of TPO‐RAs, the mice were administered 100ug kg^−1^ romiplostim (Amgen) by hypodermic injection each other day beginning on day 3. Platelet counts were measured each other day until sacrifice at 10 days after treatment. All animal studies complied with the animal care guidelines approved by the Ethics Committee of Shandong University.

### Cell Culture and Transduction

Human megakaryoblastic UT‐7 and DAMI cells were cultured and transduced as described previously.^[^ ^12^, ^56^ ^]^ In brief, these cells were cultured in 1640 medium supplemented with 10% fetal bovine serum, 100 µg mL^−1^ penicillin, and streptomycin. The culture medium of the UT‐7 cells was supplemented with erythropoietin (EPO). For ploidy analysis of DAMI cells, phorbol 12‐myristate 13‐acetate (PMA) was added to the cell culture medium for 72 h. For the TPO/c‐Mpl signaling test and flow cytometry, 50 ng mL^−1^ rhTPO or 30 µg mL^−1^ adiponectin was added.

### Plasmids

To explore proteins associated with c‐Mpl, Flag‐tagged c‐Mpl, Myc‐tagged Myosin‐9, Myc‐tagged Rgp1, and HA‐tagged Ric1 were created by PCR, subcloned, and inserted into the pcDNA3.1 vector. Rab6A and its mutant sequences were subcloned and inserted into the pEGFP‐N1 vector. Oligonucleotide siRNAs targeting Rab6A, Myosin‐9, APPL1, Rgp1and Ric1 were synthesized by GenePharma. The siRNA sequences were as follows: Rab6A‐#1: GCAGAGAAG AUAUGAUUGATT; Rab6A‐#2: CCAUUGGAAUUAUCCUUUATT; Rab6A‐#3: CUGAUAUAAAUUACGGUCUTT; Myosin‐9‐#1: GCUCCCUAAAGAACAAGCUTT; Myosin‐9‐#2: GCACUGUCAAGUCCAAGUATT; Myosin‐9‐#3: GGACCUUCCACAUCUUCUATT. APPL1‐#1: GCUGCGAUUAAUAGAUAUATT; APPL1‐#2: CGGGAGAAGUGAAAGUAAUTT; APPL1‐#3: CACGCAGGGUGGAAAUUUATT; Rgp1: CAGTGATGGCCGAGGGAAA; Ric1: GCACCTATCTAGAGAGCAA.

The shRNA sequence targeting Rab6A or Myosin‐9 was the same as the siRNA sequence.

### Immunofluorescence Staining

Bone marrow mononuclear cells (BMMCs) were collected by centrifugation and filtered through hydrophilic nylon membranes. After filtration, the cells were incubated with 1:50 dilutions of rabbit anti‐c‐Mpl (Abcam) or mouse anti‐CD41a (BD) antibodies for 1 h at room temperature and then with donkey anti‐rabbit Alexa Fluor 488 and anti‐mouse Alexa Fluor 594 secondary antibodies (Thermo Fisher Scientific) for 30 min. The samples were resuspended in 200 µL of 4% formaldehyde for 15 min to be fixed and washed in PBS. For the quantification of c‐Mpl at the cell membrane surface, UT‐7 cells transfected Flag‐c‐Mpl plasmid in which the Flag tag was inserted into the N‐terminus of c‐Mpl (the extracellular domain). After transfection, UT‐7 cells were directly stained by rabbit anti‐Flag and anti‐rabbit Alexa Fluor 488/647 without disruption of cell membrane permeability. Microscopy was performed on a Zeiss LSM 900 confocal microscope. The calculation was performed from a single optical section by the Zeiss ZEN 3.8 software.

### Flow Cytometry (FCM)

The surface expression of c‐Mpl was measured by FCM using an anti‐c‐Mpl antibody (Abcam) and an anti‐rabbit Alexa Fluor 488 secondary antibody (Thermo Fisher Scientific). Surface‐expressed CD41a was stained with an anti‐CD41a APC monoclonal antibody (BD). For UT‐7 cells, rabbit anti‐Flag and anti‐rabbit Alexa Fluor 488/647 were used. The samples were evaluated via flow cytometry (Beckman Coulter). For the polyploidy assay, cells were stained with 50 µg mL^−1^ propidium iodide (PI) (Invitrogen), 100 µg mL^−1^ RNase A, and 0.2% Triton X‐100. The samples were evaluated via FCM (Beckman Coulter).

### Western Blot and Coimmunoprecipitation (Co‐IP) Assays

Cells were lysed with TNE buffer (10 mM Tris, pH 8.0; 150 mM NaCl; 1 mM EDTA; 1% NP‐40; and 10% glycerol with protease inhibitors) after lipofection transfection or the addition of 50 ng mL^−1^ rhTPO or 30 µg mL^−1^ adiponectin. The supernatants were boiled in a loading buffer or incubated with the indicated antibody beads overnight at 4 °C. The beads were eluted by boiling in sample buffer after washing with TNE buffer six times. Then, the samples were analyzed by Western blotting with the indicated antibodies.

### Cytological Morphology Staining

After treatment, the cells were collected by centrifugation and resuspended in 200 µL of PBS. The suspension was applied to a slide and then stained with Wright–Giemsa (Sigma Diagnostics) for 5 min at room temperature. Stained cells from each treatment group were examined under a light microscope (Olympus).

### RT‐PCR Analysis

The knockdown efficiency in DAMI cells and UT‐7 cells stably transfected with shRNA lentivirus or transfected with oligonucleotide siRNAs was detected by RT‐PCR analysis. The used primer sequences are shown in Table S3 (Supporting Information).

### Statistical Analysis

Statistical analyses were performed by using two‐tailed Student's t‐tests and one‐way or two‐way ANOVA followed by Bonferroni's, Dunnett's, Tukey's or Sidak's multiple‐comparison tests, as indicated in the legends of the figures. All the data shown are presented as the means ± standard deviations (SDs), and the numbers (n) in each figure legend represent biological or technical replicates, as specified. P < 0.05 was considered to indicate statistical significance. Statistical analyses were performed using GraphPad Prism 9 (GraphPad Software).

### Ethical Statement

This study was approved by the Ethics Committee of Qilu Hospital, Shandong University. All patients and healthy donors provided informed consent in accordance with the Declaration of Helsinki.

## Conflict of Interest

The authors declare no conflict of interest.

## Author Contributions

X.Z., H.M. and G.L. contributed equally to this work. Conceptualization, H.M., X.Z., G.L., T.S., and C.J.; Supervision, T.S., and C.J.; Formal Analysis, X.Z., and H.M.; Investigation, H.M., X.Z., G.L., M.L., J.X., Z.S., T.S., and C.J.; Resources, X.L., J.X., Z.S.; Funding Acquisition, G.L., T.S., and C.J.; Writing—Original Draft, X.Z., H.M. and T.S.; Writing— Review and Editing, H.M., X.Z., G.L., X.L., T.S., and C.J.

## Supporting information



Supporting Information

## Data Availability

The data that support the findings of this study are available from the corresponding author upon reasonable request.

## References

[1] N. Cooper , W. Ghanima , I. Thrombocytopenia , The New England journal of medicine 2019, 381, 945. 31483965 10.1056/NEJMcp1810479

[2] Y. Liu , X. Zuo , P. Chen , X. Hu , Z. Sheng , A. Liu , Q. Liu , S. Leng , X. Zhang , X. Li , L. Wang , Q. Feng , C. Li , M. Hou , C. Chu , S. Ma , S. Wang , J. Peng , Signal Transduction Targeted Ther. 2022, 7, 347. 10.1038/s41392-022-01167-9 PMC9537316 36202780

[3] M. Iraqi , J. Perdomo , F. Yan , P. Y.‐I. Choi , B. H. Chong , Haematologica 2015, 100, 623. 25682608 10.3324/haematol.2014.115634 PMC4420211

[4] X. G. Liu , Y. Hou , M. Hou , J. Hematol. Oncol. 2023, 16, 4. 36658588 10.1186/s13045-023-01401-z PMC9850343

[5] S. Guillet , E. Crickx , I. Azzaoui , et al., Blood 2023, 141, 2867. 36893453 10.1182/blood.2022018665

[6] K. M. Sanfilippo , A. Cuker , Blood 2023, 141, 2790. 37289475 10.1182/blood.2023020243

[7] M. Märklin , C. Tandler , H. G. Kopp , K. L. Hoehn , L. Quintanilla‐Martinez , O. Borst , M. R. Müller , S. J. Saur , J. Cell. Mol. Med. 2020, 24, 12491. 32954656 10.1111/jcmm.15785 PMC7687000

[8] H. Al‐Samkari , D. J. Kuter , Am J Hematol 2018, 93, 1501. 30187942 10.1002/ajh.25275

[9] O. Miltiadous , M. Hou , J. B. Bussel , Blood 2020, 135, 472. 31756253 10.1182/blood.2019003599 PMC7484752

[10] M. Ballmaier , M. Germeshausen , H. Schulze , K. Cherkaoui , S. Lang , A. Gaudig , S. Krukemeier , M. Eilers , G. Strauß , K. Welte , Blood 2001, 97, 139. 11133753 10.1182/blood.v97.1.139

[11] C. Pecquet , I. Chachoua , A. Roy , T. Balligand , G. Vertenoeil , E. Leroy , R. I. Albu , J. P. Defour , H. Nivarthi , E. Hug , E. Xu , Y. Ould‐Amer , C. Mouton , D. Colau , D. Vertommen , M. M. Shwe , C. Marty , I. Plo , W. Vainchenker , R. Kralovics , S. N. Constantinescu , Blood 2019, 133, 2669. 30902807 10.1182/blood-2018-09-874578

[12] F. Basso‐Valentina , G. Levy , L. N. Varghese , M. Oufadem , B. Neven , C. Boussard , N. Balayn , C. Marty , W. Vainchenker , I. Plo , P. Ballerini , S. N. Constantinescu , R. Favier , H. Raslova , Blood 2021, 138, 480. 34010413 10.1182/blood.2020010567

[13] J. P. Defour , G. Levy , E. Leroy , S. O. Smith , S. N. Constantinescu , Leukemia 2019, 33, 563. 30635630 10.1038/s41375-018-0356-x

[14] M. Abe , K. Suzuki , O. Inagaki , S. Sassa , H. Shikama , Leukemia 2002, 16, 1500. 12145691 10.1038/sj.leu.2402554

[15] C. Pecquet , C. C. Diaconu , J. Staerk , M. Girardot , C. Marty , Y. Royer , J. P. Defour , A. Dusa , R. Besancenot , S. Giraudier , J. L. Villeval , L. Knoops , P. J. Courtoy , W. Vainchenker , S. N. Constantinescu , Blood 2012, 119, 4625. 22378845 10.1182/blood-2011-08-372524

[16] M. Schliwa , G. Woehlke , Nature 2003, 422, 759. 12700770 10.1038/nature01601

[17] L. Lordier , D. Bluteau , A. Jalil , C. Legrand , J. Pan , P. Rameau , D. Jouni , O. Bluteau , T. Mercher , C. Leon , C. Gachet , N. Debili , W. Vainchenker , H. Raslova , Y. Chang , Nat. Commun. 2012, 3, 717. 22395608 10.1038/ncomms1704

[18] J. B. Brault , S. Bardin , M. Lampic , J. A. Carpentieri , L. Coquand , M. Penisson , H. Lachuer , G. S. Victoria , S. Baloul , F. El Marjou , G. Boncompain , S. Miserey‐Lenkei , R. Belvindrah , V. Fraisier , F. Francis , F. Perez , B. Goud , A. D. Baffet , EMBO Rep. 2022, 23, 54605,. 10.15252/embr.202254605 PMC9535803 35979738

[19] Q. Chen , M. Xin , L. Wang , L. Li , Y. Shen , Y. Geng , H. Jiang , Y. Wang , L. Zhang , Y. Xu , Y. Hou , J. Liu , X. Fan , Blood 2022, 139, 2958. 35176139 10.1182/blood.2022015620

[20] S. Dahariya , S. Raghuwanshi , A. Sangeeth , M. Malleswarapu , R. Kandi , R. K. Gutti , Cancer immunology, immunotherapy : CII 2021, 70, 3477. 33890137 10.1007/s00262-021-02937-0 PMC10992152

[21] X. Zhuang , P. Xu , Y. Ou , X. Shao , Y. Li , Y. Ma , S. Qin , F. Hua , Y. Zhan , L. Ji , T. Qiao , H. Chen , Y. Cheng , Journal of translational medicine 2023, 21, 540. 37573325 10.1186/s12967-023-04389-9 PMC10423426

[22] H. T. Tzeng , Y. C. Wang , Journal of biomedical science 2016, 23, 70. 27716280 10.1186/s12929-016-0287-7 PMC5053131

[23] S. Jean , A. A. Kiger , Nature reviews. Molecular cell biology. 2012, 13, 463. 22722608 10.1038/nrm3379

[24] X. Mao , C. K. Kikani , R. A. Riojas , P. Langlais , L. Wang , F. J. Ramos , Q. Fang , C. Y. Christ‐Roberts , J. Y. Hong , R. Y. Kim , F. Liu , L. Q. Dong , Nat. Cell Biol. 2006, 8, 516. 16622416 10.1038/ncb1404

[25] X. Cai , J. Wu , Z. Y. An , et al., Br. J. Haematol. 2022, 202, 995. 36546515 10.1111/bjh.18625

[26] F. Barr , D. G. Lambright , Rab GEFs and GAPs. Current opinion in cell biology 2010, 22, 461. 20466531 10.1016/j.ceb.2010.04.007 PMC2929657

[27] G. V. Pusapati , G. Luchetti , S. R. Pfeffer , The Journal of biological chemistry 2012, 287, 42129. 23091056 10.1074/jbc.M112.414565 PMC3516758

[28] S. Siniossoglou , S. Y. Peak‐Chew , H. R. Pelham , EMBO J. 2000, 19, 4885. 10990452 10.1093/emboj/19.18.4885 PMC314221

[29] X. Ni , L. Wang , H. Wang , T. Yu , J. Xie , G. Li , Y. Liu , H. Zhou , M. Xu , M. Hou , J. Peng , Y. Hou , Blood 2022, 140, 2818. 36037415 10.1182/blood.2022016029

[30] W. Ghanima , T. Gernsheimer , D. J. Kuter , Blood 2021, 137, 2736. 33827138 10.1182/blood.2021010968

[31] G. Oberoi , M. Jain , S. M. Natu , R. Kushwaha , A. K. Tripathi , A. Kumar , Asian J Transfus Sci 2022, 16, 95. 36199407 10.4103/ajts.AJTS_65_17 PMC9528562

[32] F. Vianello , S. Vettore , F. Tezza , L. Toni , R. Scandellari , L. Sambado , M. Treleani , F. Fabris , Hematology reports 2014, 6, 4996. 24711916 10.4081/hr.2014.4996 PMC3977153

[33] M. Koupenova , A. C. Livada , C. N. Morrell , Circ. Res. 2022, 130, 288. 35050690 10.1161/CIRCRESAHA.121.319821 PMC8852355

[34] H. Zhang , B. M. Zhang , X. Guo , L. Xu , X. You , R. B. West , J. B. Bussel , J. L. Zehnder , Haematologica 2020, 105, 129. 10.3324/haematol.2019.226688 PMC7049341 31296576

[35] J. Gilleron , A. Zeigerer , Nature reviews. Endocrinology. 2023, 19, 28. 10.1038/s41574-022-00737-9 36216881

[36] F. Favale , K. Messaoudi , L. N. Varghese , S. Boukour , C. Pecquet , V. Gryshkova , J. P. Defour , R. I. Albu , O. Bluteau , P. Ballerini , G. Leverger , I. Plo , N. Debili , H. Raslova , R. Favier , S. N. Constantinescu , W. Vainchenker , Blood 2016, 128, 3146. 28034873 10.1182/blood-2016-06-722058

[37] C. Cleyrat , R. Girard , E. H. Choi , É. Jeziorski , T. Lavabre‐Bertrand , S. Hermouet , S. Carillo , B. S. Wilson , Blood advances 2017, 1, 1815. 29296828 10.1182/bloodadvances.2016002915 PMC5728092

[38] M. Banerjee , S. Joshi , J. Zhang , C. L. Moncman , S. Yadav , B. A. Bouchard , B. Storrie , S. W. Whiteheart , Blood 2017, 130, 2872. 28931526 10.1182/blood-2017-02-768176 PMC5746669

[39] N. Masubuchi , M. Araki , Y. Yang , E. Hayashi , M. Imai , Y. Edahiro , Y. Hironaka , Y. Mizukami , Y. Kihara , H. Takei , M. Nudejima , M. Koike , A. Ohsaka , N. Komatsu , Leukemia 2020, 34, 499. 31462733 10.1038/s41375-019-0564-z

[40] F. Pertuy , A. Eckly , J. Weber , F. Proamer , J. Y. Rinckel , F. Lanza , C. Gachet , C. Léon , Blood 2014, 123, 1261. 24243973 10.1182/blood-2013-06-508168

[41] K. Pal , R. Nowak , N. Billington , R. Liu , A. Ghosh , J. R. Sellers , V. M. Fowler , Blood 2020, 135, 1887. 32315395 10.1182/blood.2019003064 PMC7243143

[42] E. Del Nery , S. Miserey‐Lenkei , T. Falguières , C. Nizak , L. Johannes , F. Perez , B. Goud , Traffic 2006, 7, 394. 16536738 10.1111/j.1600-0854.2006.00395.x

[43] A. Petrosyan , C. A. Casey , P. W. Cheng , Sci. Rep. 2016, 6, 31962. 27535804 10.1038/srep31962 PMC4989220

[44] A. Sharma , M. Mah , R. H. Ritchie , M. J. De Blasio , Pharmacol. Ther. 2022, 232, 108008. 34610378 10.1016/j.pharmthera.2021.108008

[45] S. Sun , W. Wang , Y. Latchman , D. Gao , B. Aronow , J.‐A. Reems , Physiological genomics 2013, 45, 217. 23321270 10.1152/physiolgenomics.00056.2012 PMC3615580

[46] X. H. Zhou , Z. P. Cheng , M. Lu , W.‐Y. Lin , L.‐L. Luo , Z. Y. Ming , Y. Hu , Acta Pharmacol. Sin. 2023, 44, 356. 35918410 10.1038/s41401-022-00943-1 PMC9889809

[47] C. Valet , A. Batut , A. Vauclard , et al., Adipocyte Fatty Acid Transfer Supports Megakaryocyte Maturation. Cell reports. 2020, 32, 107875. 32640240 10.1016/j.celrep.2020.107875

[48] J. Cao , L. Ji , Y. Zhan , X. Shao , P. Xu , B. Wu , P. Chen , L. Cheng , X. Zhuang , Y. Ou , F. Hua , L. Sun , F. Li , H. Chen , Z. Zhou , Y. Cheng , Cell Mol. Immunol. 2023, 20, 1413. 37833401 10.1038/s41423-023-01089-8 PMC10687271

[49] K. Ohashi , J. L. Parker , N. Ouchi , A. Higuchi , J. A. Vita , N. Gokce , A. A. Pedersen , C. Kalthoff , S. Tullin , A. Sams , R. Summer , K. Walsh , J. Biol. Chem. 2010, 285, 6153. 20028977 10.1074/jbc.M109.088708 PMC2825410

[50] H. Xu , Q. Zhao , N. Song , Z. Yan , R. Lin , S. Wu , L. Jiang , S. Hong , J. Xie , H. Zhou , R. Wang , X. Jiang , Nat. Commun. 2020, 11, 5807. 33199780 10.1038/s41467-020-19668-y PMC7669869

[51] R. C.‐L. Ng , M. Jian , O. K.‐F. Ma , M. Bunting , J. S.‐C. Kwan , G. J. Zhou , K. Senthilkumar , A. Iyaswamy , P. K. Chan , M. Li , K. M.‐Y. Leung , S. S. Kumar Durairajan , K. S.‐L. Lam , L. W. Chu , R. Festenstein , S. K. Chung , K.‐H. Chan , Mol. Psychiatry 2021, 26, 5669. 32132650 10.1038/s41380-020-0701-0

[52] I. Gianopoulos , C. S. Mantzoros , S. S. Daskalopoulou , Endocr Rev 2025, 46, 1. 39106421 10.1210/endrev/bnae021 PMC11720176

[53] D. V. Pham , P. H. Park , J Exp Clin Cancer Res 2022, 41, 9. 34986886 10.1186/s13046-021-02223-y PMC8729140

[54] D. Provan , D. M. Arnold , J. B. Bussel , B. H. Chong , N. Cooper , T. Gernsheimer , W. Ghanima , B. Godeau , T. J. González‐López , J. Grainger , M. Hou , C. Kruse , V. McDonald , M. Michel , A. C. Newland , S. Pavord , F. Rodeghiero , M. Scully , Y. Tomiyama , R. S. Wong , F. Zaja , D. J. Kuter , Blood Adv 2019, 3, 3780. 31770441 10.1182/bloodadvances.2019000812 PMC6880896

[55] F. Rodeghiero , R. Stasi , T. Gernsheimer , M. Michel , D. Provan , D. M. Arnold , J. B. Bussel , D. B. Cines , B. H. Chong , N. Cooper , B. Godeau , K. Lechner , M. G. Mazzucconi , R. McMillan , M. A. Sanz , P. Imbach , V. Blanchette , T. Kühne , M. Ruggeri , J. N. George , Blood 2009, 113, 2386. 19005182 10.1182/blood-2008-07-162503

[56] M. H. Laprise , F. Grondin , P. Cayer , P. P. McDonald , C. M. Dubois , Blood 2002, 100, 3578. 12411321 10.1182/blood.V100.10.3578

