# Supplementary material for: Adiponectin Assists Thrombopoietic Agents in ITP Treatment by Enhancing Myosin‐9/Rab6A‐Mediated Trafficking of c‐Mpl in MKs
Source: Adv Sci (Weinh). 2025 Jun 25;12(34):e03008. doi: 10.1002/advs.202503008 (PMC12442642; doi:10.1002/advs.202503008)
Supplement: Supplementary file 1 — Supporting Information [file ADVS-12-e03008-s001.docx]

Supporting Information

**Adiponectin Assists Thrombopoietic Agents in ITP Treatment by Enhancing Myosin-9/Rab6A-Mediated Trafficking of c-Mpl in MKs**

Xin Zhao^#^, Huixian Ma^#^, Guosheng Li^#^, Jilong Xiao, Zhiyu Shi, Mingying Li, Xinguang Liu, Tao Sun*, Chunyan Ji*

**Supplementary figure 1**

**
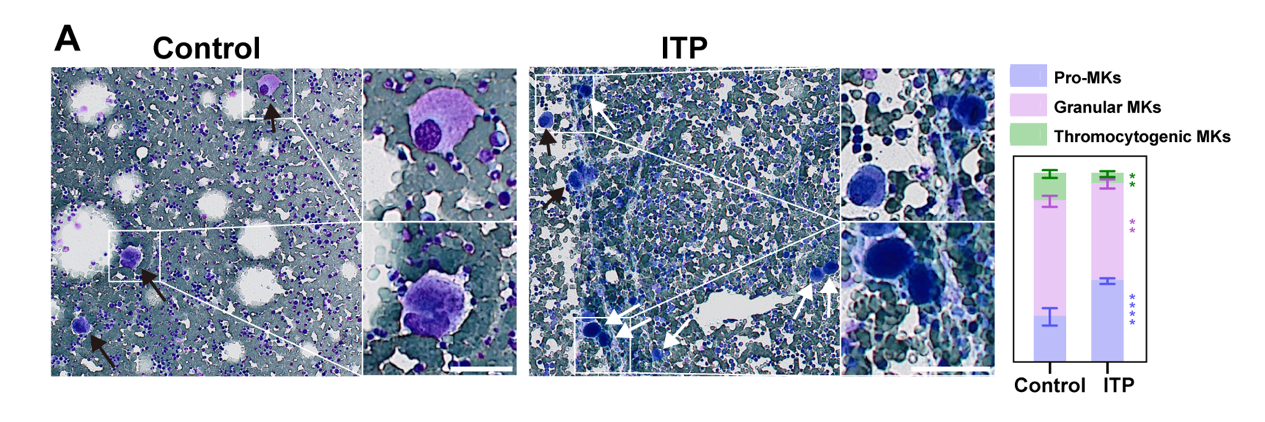
**

**Figure S1. The bone marrow aspiration from healthy control and ITP patients.**

(A) The representative images and the quantitative summary of bone marrow aspiration from healthy control and ITP patients, in which megakaryocytes are amplified on the right. White arrows point to pro-MKs, short black arrows point to granular MKs and long black arrows point to thromocytogenic MKs. **P < 0.01; ****P < 0.0001. Mean ± SD values are shown.

**Supplementary figure 2**

**
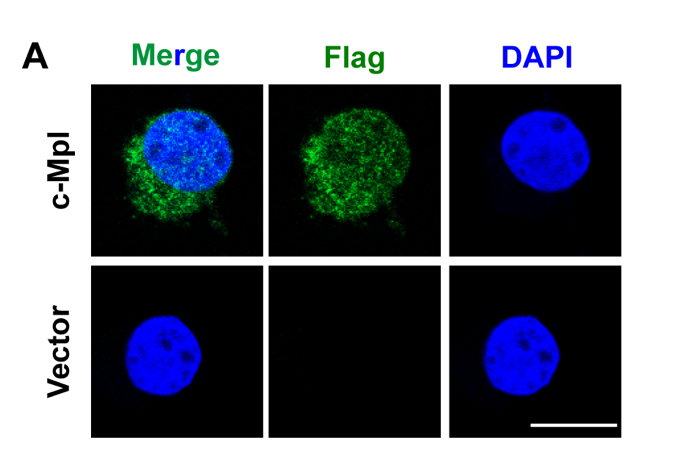
**

**Figure S2. The transfected control of Flag-c-Mpl plasmid.**

(A)The representative images of UT-7 cells transfected with empty vector and Flag-c-Mpl plasmid in which the Flag tag was inserted into the N-terminus of c-Mpl (the extracellular domain). After transfection, UT-7 cells were directly stained by rabbit anti-Flag and anti-rabbit Alexa Fluor 488 without disruption of cell membrane permeability.

**Supplementary figure 3**


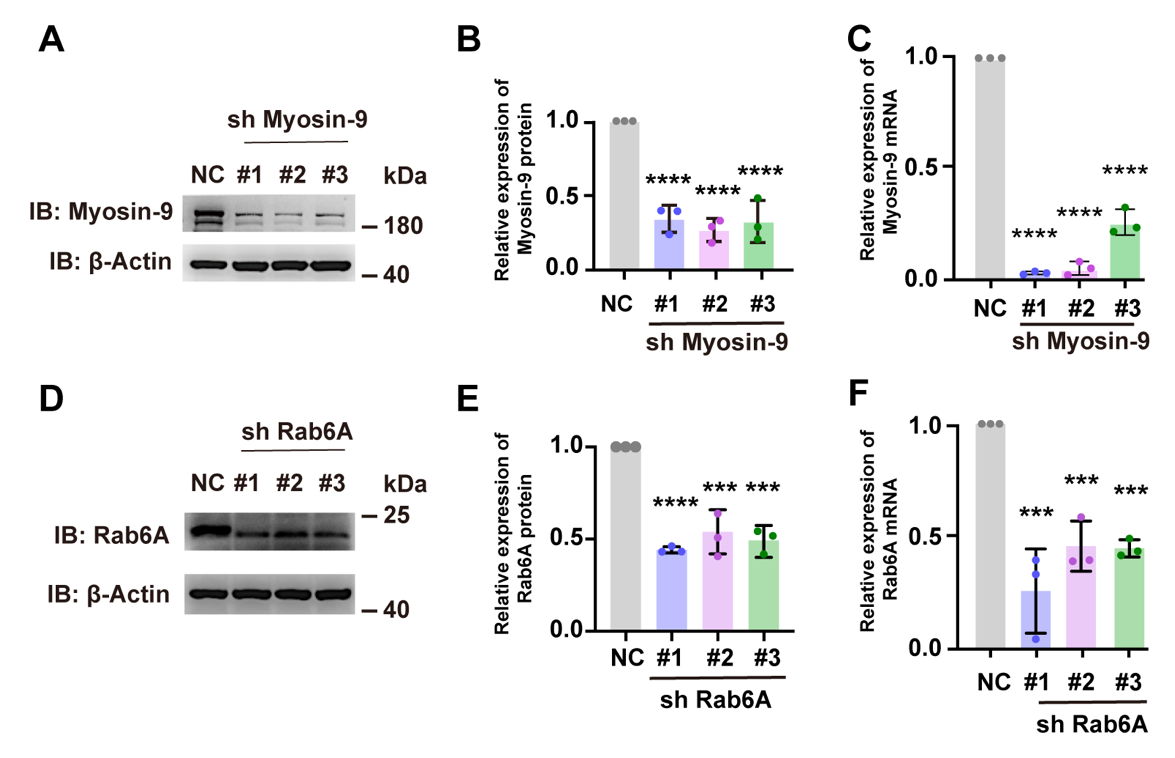


**Figure S3. The interference efficiency was verified by RT‒PCR and Western blotting.**

(A-C) The protein expression (A-B) and relative mRNA expression (C) of Myosin-9 in shMyosin-9 DAMI cells (n=3). ****P < 0.0001. (D-F) The protein expression (D-E) and relative mRNA expression (F) of Rab6A in shRab6A DAMI cells (n=3). ***P < 0.001; ****P < 0.0001. Mean ± SD values are shown for the statistical analysis.

**Supplementary figure 4**

**
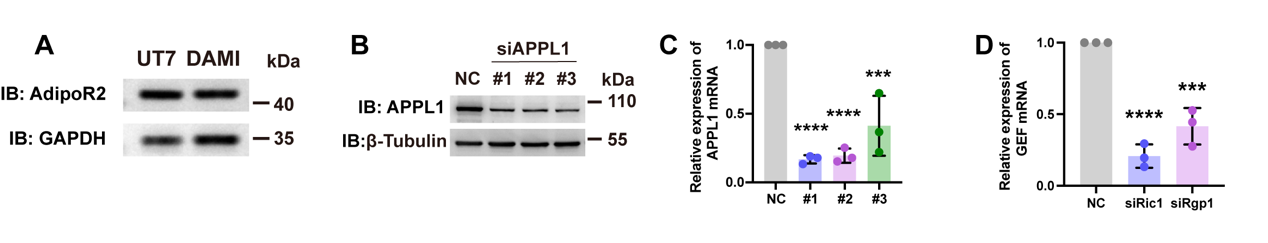
**

**Figure S4. The expression and interference efficiency were verified by RT‒PCR and Western blotting.**

(A) The protein expression of AdipoR2 in UT7 and DAMI cells. (B-C) The protein expression (B) and relative mRNA expression (C) of APPL1 in siAPPL1 HEK-293T cells (n=3). ***P < 0.001; ****P < 0.0001. (D) The relative mRNA expression of Ric1 or Rgp1 in siRic1 or siRgp1 HEK-293T cells (n=3). ***P < 0.001; ****P < 0.0001. Mean ± SD values are shown for the statistical analysis.

**Supplementary figure 5**

**
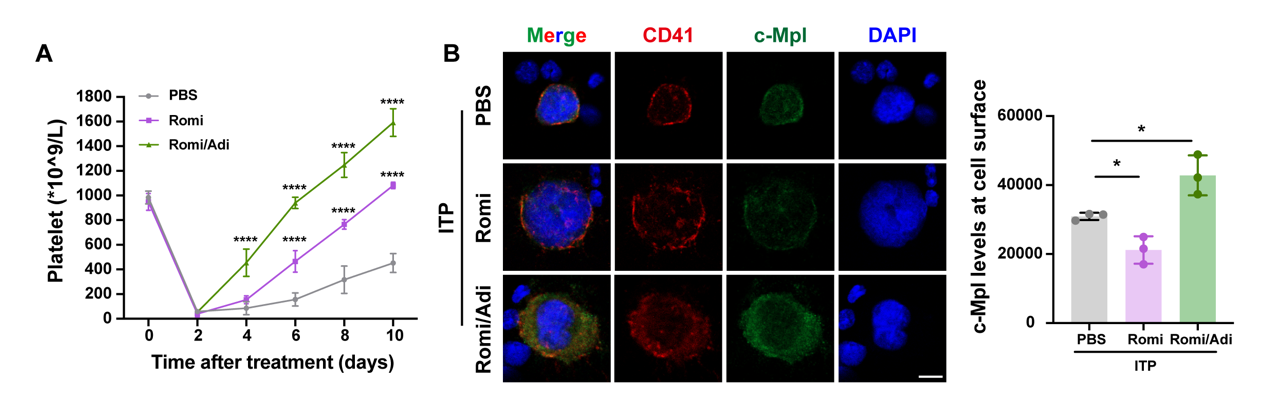
**

**Figure S5. Adiponectin combined with Romiplostim increase therapeutic effects in ITP mice.**

(A) Peripheral blood platelet counts of mice with different treatment were monitored at different time points (n=5). ****P < 0.0001. (B-C) Representative confocal microscopy images and statistical analysis show the expression of c-Mpl on MKs surface from bone marrow of the mice with different treatment. At least 15 MK cells were quantified per experimental group (n=3). CD41a, red; c-Mpl, green. Scale bars, 10μm. *P < 0.05. Mean ± SD values are shown.

**Supplementary figure 6**


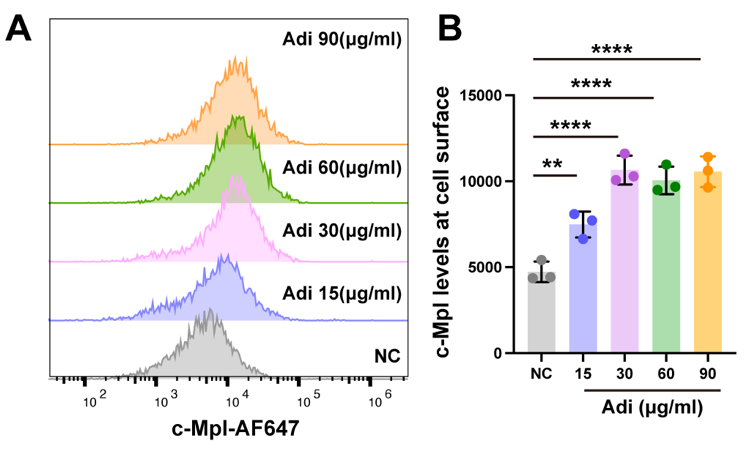


**Figure S6. Adiponectin enhanced the trafficking of c-MPL to the membrane in a control manner**

(A&B) Representative histograms and statistical analysis of c-Mpl expression on surface of NC and adiponectin treated UT-7 cells determined via flow cytometry. The geometric mean fluorescence intensity is shown(n=3). **P < 0.01; ****P < 0.0001. Mean ± SD values are shown.

**Table S1 | Baseline characteristics of ITP patients and healthy controls**

|  | Healthy controls (N = 14) | Treatment-naïve ITP patients (N = 38) | rhTPO/TPO-RAs resistant ITP patients (N = 19) | *P* |
| --- | --- | --- | --- | --- |
| Age | 41.50（23-56） | 47.00（19-75） | 56.00（26-80） | 0.123 |
| Gender |  |  |  |  |
| Male | 9（64.3） | 17（44.7） | 8（42.1） | 0.384 |
| Female | 5（35.7） | 21（55.3） | 11（57.9） | 0.384 |
| Baseline platelet count（×10^9^/L) | 231（187-291） | 3（0-28） | 3（0-23） | ＜0.001 |
| Previous therapies, n | NA | 0 | 3（2-5） | NA |

^Data are median (range) or n (%). NA, not applicable.^

**Table S2 | Information of enrolled rhTPO/TPO-RAs resistant^*^ primary ITP patient**

| No. | Age | Gender | Baseline platelet count（×10^9^/L) | Previous therapies |
| --- | --- | --- | --- | --- |
| 1 | 42 | Male | 1 | Corticosteroids and TPO-RAs |
| 2 | 74 | Female | 1 | Corticosteroids, IVIg, TPO-RAs and rhTPO |
| 3 | 39 | Male | 2 | Corticosteroids, Rituximab, TPO-RAs and rhTPO |
| 4 | 27 | Male | 15 | Corticosteroids, TPO-RAs and rhTPO |
| 5 | 57 | Female | 8 | Corticosteroids, IVIg, TPO-RAs and rhTPO |
| 6 | 66 | Female | 4 | Corticosteroids, IVIg and rhTPO |
| 7 | 59 | Female | 4 | Corticosteroids, IVIg, Rituximab and rhTPO |
| 8 | 65 | Female | 3 | Corticosteroids and TPO-RAs |
| 9 | 30 | Female | 3 | Corticosteroids, IVIg and rhTPO |
| 10 | 60 | Male | 0 | Corticosteroids and TPO-RAs |
| 11 | 80 | Male | 6 | Corticosteroids and rhTPO |
| 12 | 44 | Male | 1 | Corticosteroids and TPO-RAs |
| 13 | 65 | Female | 23 | Corticosteroids and TPO-RAs |
| 14 | 40 | Male | 2 | Corticosteroids, IVIg and rhTPO |
| 15 | 56 | Female | 15 | Corticosteroids, Rituximab and rhTPO |
| 16 | 26 | Female | 3 | Corticosteroids, Rituximab, TPO-RAs and rhTPO |
| 17 | 53 | Female | 11 | Corticosteroids and TPO-RAs |
| 18 | 65 | Male | 12 | Corticosteroids, TPO-RAs and rhTPO |
| 19 | 32 | Female | 1 | Corticosteroids, IVIg, Rituximab, TPO-RAs and rhTPO |

^*^ ^Corticosteroids have been given to the patient and at least one of the treatments was rhTPO injection or TPO-RAs.^

**Table S3 | q-PCR primer sequences**

| Primer | Sequence (5' to 3' direction) |
| --- | --- |
| Homo-Myosin-9-F | CGCGAAGTCAGCTCCCTAAA |
| Homo-Myosin-9-R | GCCATCTACCTCTTCGTCGG |
| Homo-Rab6A-F | CGGGAATGGAAAGCACACAG |
| Homo-Rab6A-R | CACTGACTGGTTGCTCCTGA |
| Mus-Rgp1-F | CTGTCTTTCTGCCACACCGA |
| Mus-Rgp1-R | GACTGACTGACCCCGAAAGG |
| Mus-Ric1-F | GGTACAGCCGACCTAGTGTG |
| Mus-Ric1-R | CCATTTGCTGTTGACACTGCT |
| Homo-APPL1-F | TCCAGTAGACCAAACCAAGCC |
| Homo-APPL1-R | TCTCTTCTGACTGGCTACTGC |
| Homo-Rgp1-F | AGAGGGGCCAGTGTATCCTT |
| Homo-Rgp1-R | CCCTCTATGGGCAGCACTTC |
| Homo-Ric1-F | CATTCCCGACCCTCTGCTTC |
| Homo-Ric1-R | GGTCTTCCTGGCACAATGGA |
| Mus-AdipoR2-F | CCACACGAGTTACCAATGAC |
| Mus-AdipoR2-R | GTTCTGGGGATTTCTTGGTC |
| Homo-AdipoR2-F | GAAAACCGATTGGGGTGCAG |
| Homo-AdipoR2-R | TGGGCTCCAAATCTCCTTGG |
